# Development and Validation of a Radiomics Nomogram for Prognosis Prediction of Patients with Acute Paraquat Poisoning: A Retrospective Cohort Study

**DOI:** 10.1155/2021/6621894

**Published:** 2021-02-02

**Authors:** Shan Lu, Duo Gao, Yanling Wang, Xuran Feng, Yongzhi Zhang, Ling Li, Zuojun Geng

**Affiliations:** Department of Medical Imaging, The Second Hospital of Hebei Medical University, Shijiazhuang 050000, China

## Abstract

**Objective:**

To evaluate the efficiency of a radiomics model in predicting the prognosis of patients with acute paraquat poisoning (APP).

**Materials and Methods:**

Chest computed tomography images and clinical data of 80 patients with APP were obtained from November 2014 to October 2017, which were randomly assigned to a primary group and a validation group by a ratio of 7 : 3, and then the radiomics features were extracted from the whole lung. Principal component analysis (PCA) and least absolute shrinkage and selection operator (LASSO) regression were used to select the features and establish the radiomics signature (Rad-score). Multivariate logistic regression analysis was used to establish a radiomics prediction model incorporating the Rad-score and clinical risk factors; the model was represented by nomogram. The performance of the nomogram was confirmed by its discrimination and calibration.

**Result:**

The area under the ROC curve of operation was 0.942 and 0.865, respectively, in the primary and validation datasets. The sensitivity and specificity were 0.864 and 0.914 and 0.778 and 0.929, and the prediction accuracy rates were 89.5% and 87%, respectively. Predictors included in the individualized predictive nomograms include the Rad-score, blood paraquat concentration, creatine kinase, and serum creatinine. The AUC of the nomogram was 0.973 and 0.944 in the primary and validation datasets, and the sensitivity and specificity were 0.943 and 0.955, respectively, in the primary dataset and 0.889 and 0.929 in the validation dataset, and the prediction accuracy was 94.7% and 91.3%, respectively.

**Conclusion:**

The radiomics nomogram incorporates the radiomics signature and hematological laboratory data, which can be conveniently used to facilitate the individualized prediction of the prognosis of APP patients.

## 1. Introduction

Although some countries have banned the use of paraquat (PQ), paraquat can still be obtained on the market by other forms of preparations. In recent years, the incidence rate of paraquat poisoning is still high in some areas of China, and paraquat poisoning has become the first cause of death of poisoning. After ingestion, PQ is rapidly absorbed and distributed to the lung, liver, kidney, and muscle, and if left untreated, the accumulation of PQ can cause fulminant multiple organ failure, including pulmonary edema and heart, kidney, and liver failure [1], with a mortality rate of up to 50%~90% [2]. However, there is still no effective antidote. PQ mainly accumulates in the lung, where it is retained even when blood levels start to decrease, resulting in a free radical build-up that triggers inflammatory responses and leading to lung fibrosis [3, 4]. Lung damage and respiratory failure are common causes of death [5, 6]. Although many studies suggested that the lung injury caused by paraquat is irreversible, a case study, in fact, by Lee et al. [7] showed that lung damage may not be irreversible if treated in time. Thus, evaluation of lesions in the lungs and their severity at the early stage of poisoning may be crucial to guide the clinical adjustment of the treatment plan and improve patient outcomes.

Chest computed tomography (CT) has been demonstrated to be useful in detecting early lung lesions and assessing long-term damage in PQ-poisoned survivors [1]. In the 1990s, Im et al. [8] and Lee et al. [4] described the radiologic high-resolution CT (HRCT) manifestations of PQ-induced pulmonary damage, with special emphasis on the sequential changes, but without quantitative studies. Recently, the number of injured lung segments and the volume or area ratio of gross glass density shadow (GGO) found in CT examination in patients with acute PQ poisoning have been used to predict the prognosis of PQ poisoning [5, 6, 9]; although these studies obtained a certain accuracy rate, their observation object was limited to a single injury sign, which lacked estimation of the total lung injury and ignored a large part of the CT image information.

**Figure 1:**
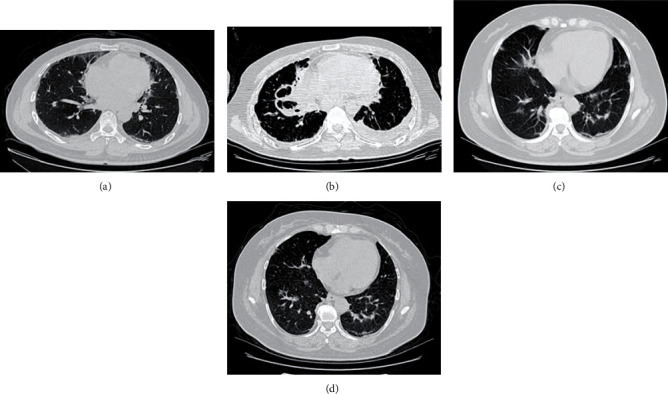
(a, b) CT images of paraquat poisoning in a 53-year-old man at the beginning and 2 months later. (c, d) CT images of paraquat poisoning in a 40-year-old woman at the beginning and 2 months later.

Although studies have shown that many blood laboratory indicators can also be used to predict the prognosis of patients with PQ poisoning [10–17], most of these studies used only one or several indicators that were almost lung nonspecific, which cannot effectively reflect the major causes of death of APP patients: injury of the lung. A comprehensive predictive model, which combines CT lung injury signs and blood laboratory indicators, to evaluate multisystem injury or functional failure is yet to be developed. Previous studies have also shown that objective and quantitative imaging descriptors could potentially be used as prognostic or predictive biomarkers. The combined analysis of a panel of biomarkers, rather than individual analyses, as a signature is the most promising approach that is powerful enough to change clinical management [18–20]. As is shown in Figure 1, the two patients had similar lung damage at the initial stage of poisoning. However, CT examination showed that the severity of pulmonary disease was different after 2 months of follow-up so as the prognosis. Radiomics, as one of the most representative methods, is the process of the conversion of medical images into high-dimensional, mineable data via high-throughput extraction of quantitative features, followed by subsequent data analysis for decision support, which has been demonstrated useful in many kinds of focal lesions [21, 22]. However, to our best knowledge, rare radiomics applications for diffuse lesions were reported yet.

Therefore, the purpose of this study is to explore the feasibility of radiomics for the study of diffuse inflammatory diseases and to develop and validate a nomogram based on CT radiomics features and clinical prognostic risk factors for predicting the prognosis of patients with APP.

## 2. Patients and Methods

This study was approved by the Hospital Ethics Committee, and the requirement for written informed consent was waived.

### 2.1. Participants

**Figure 2:**
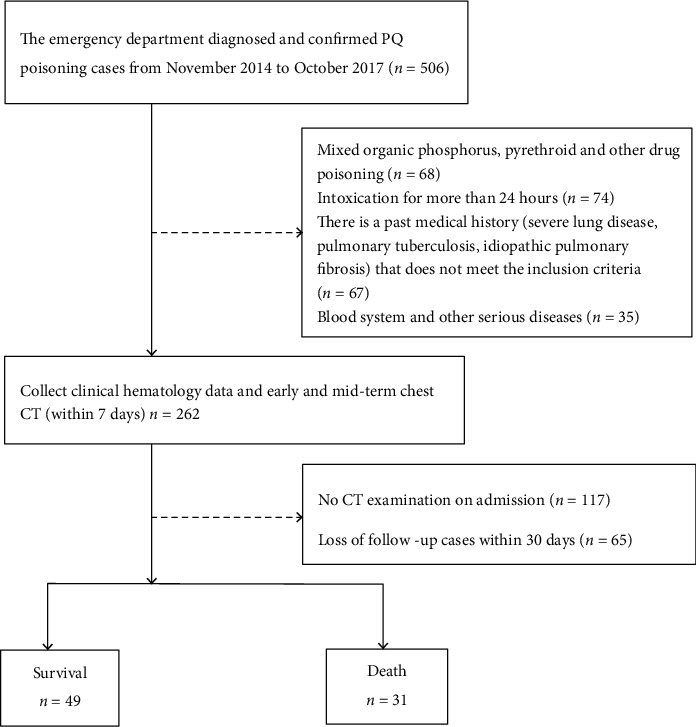
Flow chart of study enrollment.

Initial clinical baseline data and CT examination images of acute paraquat-poisoned patients, who were admitted to the emergency department from November 2014 to October 2017 and received individualized comprehensive treatment (Data Supplement (available here)), were collected. The patient screening process is shown in Figure 2. Data Supplement presents the inclusion and exclusion criteria.

The initial clinical baseline data of the poisoned patients included the following: age, gender, PQC, and blood routine and biochemical indicators within 24 hours, which included white blood cell count (WBC), high-sensitivity C-reactive protein (hsCRP), lactate dehydrogenase (LDH), creatine kinase isoenzyme (CK-MB), alanine aminotransferase (ALT), aspartate aminotransferase (AST), albumin (ALB), urea, creatinine (Cr), amylase (AMY), and glucose (GLU), a total of 12 indicators.

Finally, 80 patients were included in the study. According to the follow-up outcome of 30 days after PQ ingestion, patients were divided into the survival group (>30 days) and the death group. All 80 patients were randomly divided into two groups according to a ratio of 7 : 3.

### 2.2. CT Image Acquisition

Chest CT examinations were performed using a GE LightSpeed/16-slice scanner. CT scanning parameters were the same as those of the chest: 120 kV, 100 mA, 5 mm thickness and slice interval, and standard lung window (window width, 1500 HU; window level, -700 HU) were selected. Within 7 days after taking PQ, a chest CT examination was performed every average of 3 days.

### 2.3. Image Segmentation: ROI Drawing Methods and Modification Criteria

**Figure 3:**
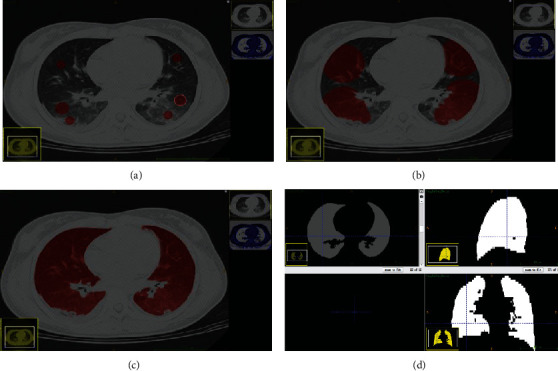
Image segmentation diagram. (a) Seed points were selected in three higher density lesions, lower density lesions, and normal lung tissue in both lungs. (b) The seed points began to grow. (c) Growth of seed points completed basically. (d) ROI obtained after manual modification.

We used the region growing method in the ITK-SNAP software (version 3.6.0, https://www.itksnap.org) to sketch the whole lung as the ROI, which was then manually modified by two physicians with licensed physician qualifications. The interobserver correlation coefficients (ICCs) were used to assess the agreement of radiomics features by two-level radiologists. Data Supplement presents the ROI drawing methods and modification criteria in Figure 3.

The region growing method is mainly divided into three steps. (1) The seed points were selected from the seed area that can represent the extraction area, and the seed was a small area including a couple of pixels. (2) Determine the criteria for region growing and measure whether the pixels adjacent to the seed point meet the criteria. The standards outlined in this study were as follows: the lower threshold was -1200 HU, and the upper threshold was -100 HU. (3) Stop growing [18]. After region growing, the boundary between the apex of the lung and the edge of the lung needed manual modification.

The criteria for manual modification were as follows. (1) In the boundary between the chest wall and the lung, the lesion-free areas were automatically outlined without modification; those areas with lesions (but the lesions were not totally included in the ROI) were manually modified. (2) If the demarcation of lung atelectasis caused by pleural effusion was unclear, automatic delineation of results was used without manual modification. (3) For the higher density of lung lesions, such as cords and nodules, which were not covered in the ROI, manual delineation was applied. (4) For lung lesions that were not included in the ROI automatically, manual delineation was applied. (5) For the vascular and bronchi, the ROI contained no main and leaf bronchi; if the segmental and inferior bronchi were connected to pixels that were distinguishable by the naked eye, we did not sketch them into the ROI. Otherwise, we sketched them into the ROI; the small scattered bronchus of lungs was contained in the ROI. (6) For those lesions with a poor borderline in the hilum, the principle was not missing lesions as far as possible. (7) For the apex and bottom of the lung, slices without lung tissue were removed manually; slices with lung tissue but only scattered pixels in the border were included; we modified the ROI to the edge of the lung tissue manually.

### 2.4. Radiomics Feature Extraction

Analysis Kit software (GE Healthcare, Life Sciences, China) was utilized to extract the radiomics features. A total of 385 radiomics features, including 42 histogram features, 154 grey-level cooccurrence matrix (GLCM) features, 180 run-length matrix (RLM) features, and 11 grey-level zone size matrix (GLZSM) features, were extracted from the ROI. Details of the radiomics feature extraction methodology and the individual parameters can be found in the Data Supplement. The interobserver correlation coefficient (ICC) between two radiologists' agreement is 0.823 (0.762 to 0.971, 95% CI).

### 2.5. Feature Selection and Radiomics Signature Building

The principal component analysis (PCA) and the least absolute shrinkage and selection operator (LASSO) method were used to select the most useful predictive features from the primary cohort. A radiomics signature (here we called the Rad-score) was calculated for each patient via a linear combination of selected features that were weighted by their respective coefficients.

### 2.6. Radiomics Signature Validation

We evaluated the ability of the Rad-score to differentiate survival and death in the primary cohort and then validated it in the validation cohort. Sensitivity, specificity, and AUC (area under the ROC curve) were used to evaluate the diagnostic efficiency. The diagnostic accuracy rate was shown as a color bar chart.

### 2.7. Development of an Individualized Prediction Model

Statistical analysis and ROC curve analysis were performed for each initial clinical baseline data, and backward logistic regression was used to select clinical risk factors to be included in the nomogram.

An individualized prediction model was established based on the primary dataset by incorporating the radiomics signature with the clinical risk factors. And it was presented with a radiomics nomogram so as to provide the clinicians with a quantitative tool to predict prognosis. Calibration curves were plotted to assess the calibration of the radiomics nomogram. Decision curve analysis (DCA) was conducted to determine the clinical usefulness of the radiomics nomogram by quantifying the net benefits at different threshold probabilities in the testing dataset.

### 2.8. Statistical Analysis

Statistical analyses were performed by using SPSS 21.0.*P* < 0.05 was considered statistically significant. A chi-squared test was used for the comparison of count data. Measurement data were compared by using the independent-samples *t*-test if the data satisfied the normal distribution, otherwise by using the Mann-Whitney *U* test. Measurement data were generally expressed as the mean ± standard deviation or the median and the interquartile range according to whether satisfying normal distribution.

Feature selection and model building were conducted with R software (version 3.3.2; http://www.Rproject.org).

## 3. Results

### 3.1. Clinical Risk Factor Selection

The statistical test results of demography and initial blood laboratory data and are shown in Table 1. The results showed that PQA, PQC, WBC, CK-MB, LDH, Cr, and GLU were statistically significant among the survival and death groups (*P* < 0.05), and the ROC curve showed AUC of PQA, PQC, WBC, CK-MB, and Cr were all above 0.7. Finally, PQC, CK-MB, and Cr were selected by backward logistic regression to be included in the nomogram.

### 3.2. Feature Selection and Radiomics Signature Building

**Figure 4:**
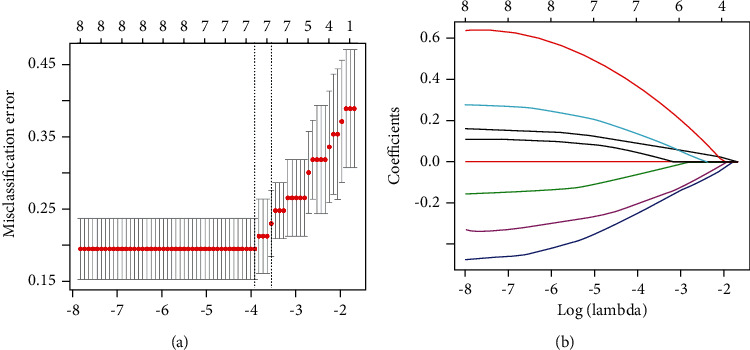
The number of features that LASSO selected after cross-validation. The underlined part is the value of log (lambda) and the number of features when the misclassification error is minimum.

Among the 385 original features from the primary dataset extracted, 23 constant terms were deleted first; 8 features with a cumulative variance contribution rate of 95% were retained after PCA (Appendix Figure A1 is given in the Data Supplement). The seven most relevant features were finally selected using LASSO, which gave the minimum mean classification error of cross-validation (Figures 4(a) and 4(b)).

### 3.3. Diagnostic Validation of the Radiomics Signature

**Figure 5:**
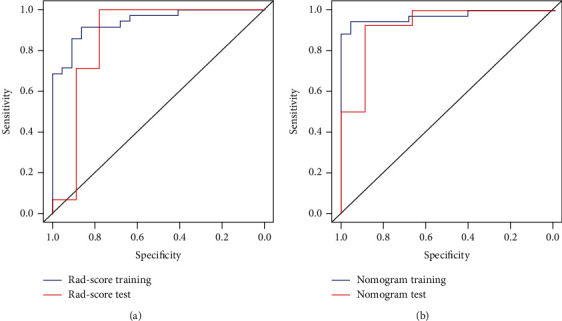
The ROC curve for the Rad-score and nomogram of the primary dataset and validation dataset.

ROC curves were plotted to evaluate the diagnostic efficiency of the logistic regression models (Figure 5(a)). The accuracy of the Rad-score is shown in Table 2. Distributions of the Rad-score and prognosis status in the primary and validation cohorts are given in the Data Supplement Appendix Figure A2.

### 3.4. Development of Individualized Prediction Comprehensive Models

**Figure 6:**
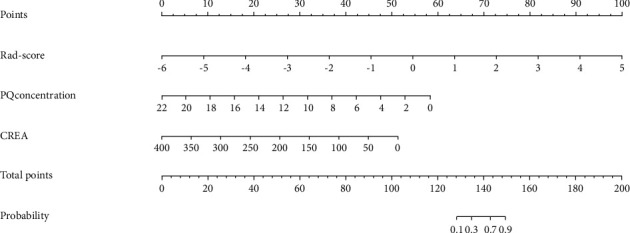
Radiomics nomogram was developed in the primary dataset.

Incorporated clinical factors included PQC, CK-MB, and Cr with the Rad-score; using multivariable logistic regression analysis, an individualized prediction model was built and is shown as a nomogram in Figure 6. The ROC curves were plotted to evaluate the diagnostic efficiency of the comprehensive model and are shown in Figure 5(b) and Table 2.

### 3.5. Clinical Use

**Figure 7:**
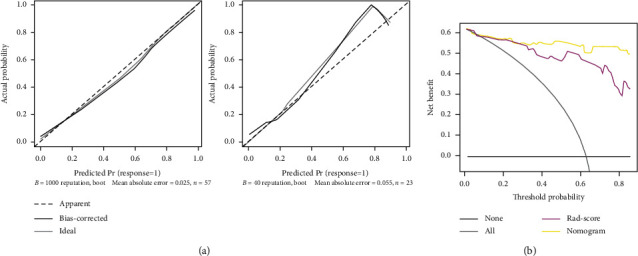
(a) Calibration curves of the radiomics nomogram. (b) Decision curve analysis for the radiomics nomogram.

The calibration curves of the primary dataset and validation dataset showed good agreement between prediction probability and real probability (Figure 7(a)). The decision curve showed that if the threshold probability of a patient or doctor is >10%, using the Rad-score to predict the prognosis of the patients adds more benefit than either the treat-all-patients scheme or the treat-none scheme. If the threshold probability exceeds 30%, the nomogram combining the Rad-score and clinical risk factors will be the best choice to maximize the net benefit (Figure 7(b)).

## 4. Discussion

Our study results revealed 385 radiomics features of pulmonary CT images, and we reduced them to 7 potential predictors and established the radiomics signature. The AUC of the primary dataset and validation dataset, respectively, were 0.942 (95% CI 0.886-0.997) and 0.865 (95% CI 0.658-1), and the sensitivity and specificity, respectively, were 0.864 and 0.914 and 0.778 and 0.929. The prediction accuracy of primary and validation datasets was 89.5% and 87%, respectively, which showed that the Rad-score had a good performance in the prediction of patient prognosis.

In previous studies about prognosis based on the pulmonary CT, Zhang et al. [5] found significantly fewer involved lung segments, or the presenting lesions were observed in baseline CT images (average admission 2.4 days) from the survivor group than the nonsurvivor group, indicating a smaller baseline disease extent in surviving patients. In their study, the sensitivity and specificity to predict prognosis were 72.2% and 28.6%, respectively, and the AUC was 0.767 (95% CI 0.656-0.878), based on the number of injured lung segments in the baseline CT examination. Their sensitivity and specificity were not very high for patient prognostic evaluation. Kim et al. [9] calculated the ratio of the sum of the areas of GGO at five levels (the top of the aortic arch, AP window, LUL bronchus, right inferior vein, and the top of the left diaphragm, respectively) and the sum of the area of the total lungs at the respective levels of pulmonary HRCT images 7 days after PQ ingestion, thinking that the area of GGO in the lung was an additional useful predictor for survival, especially when the PQ level was low. Kang et al. [6] calculated the maximum GGO volume ratio to the whole lung within the first 5 days after intoxication and showed that the AUC was 0.871 (95% CI 0.857-0.884), the sensitivity was 85.4%, the specificity was 89.3%, and the diagnostic accuracy was 87.6%. However, their study lacked independent validation; thus, the reliability of the obtained results needed to be further studied. Early lung injury of PQ intoxication mainly manifested as alveolitis, which was often shown as GGO and consolidation in pulmonary CT images. Therefore, GGOs could reflect a certain extent of lung injury. The relatively accurate results of previous studies proved that the range of lung injury was an important factor for patient prognosis. However, the number of injured lung segments, GGO area ratio or volume ratio, could not completely reflect the extent of lung injury involving the whole lung and neglected other lung injuries that were not easily quantified, such as the thickening of bronchovascular bundles. In addition, all the GGO lesions in their study were manually delineated, resulting in large errors and poor consistency; and regarding calculating the area ratio or volume ratio, lesions and whole lungs needed to be delineated twice or even repeatedly examined. Not only was the work inefficient, but it also further increased the error.

In this study, the region growing method was used to semiautomatically delineate the ROI. The whole lung was selected as the ROI of the CT images that the lung injury reached the peak (mainly 2-4-day images). Not only did it cover all the signs of lung injury we observed, but it was also easier to study ubiquitous lesions that are difficult to quantify, such as the thickening of the bronchovascular bundle. This provided a comprehensive measure of the extent and severity of lung injury, which would not ignore the microstructure changes that were invisible to naked eyes. Moreover, the more injury signs were observed in the same image, the more rapidly the lung injury developed, so the whole lung was selected as the ROI and was more scientific and rigorous.

In the early stage of lung injury caused by PQ poisoning, CT image mainly manifested as lung texture enhancement, GGO or consolidation, and was mainly distributed under the pleura. The features of density, range, and distribution of the above lung injuries may be the response of microstructural changes, including cell morphological changes and apoptosis, alveolar rupture and alveolar collapse, vascular basement membrane rupture, fibroblast precursor proliferation, and Clara cell migration [3, 23–27]. The Rad-score calculated based on the radiomics features that were extracted from CT images can effectively distinguish the different prognoses of the patients; thus, we guess that the radiomics features, such as the first-order histogram features and texture features, not only reflected the visible injuries by the naked eyes but also suggested the changes of the lung microstructure.

Among the laboratory data obtained at presentation, the levels of potassium, protein, arterial pH, PaCO_2_, bicarbonate, albumin, amylase, AST, BUN, creatinine, and glucose were significantly related with prognosis by univariable analysis in a previous study [17]. However, among many similar studies, the strength of the correlation of various indicators with prognosis was different, which may be explained by the different equipment used, the follow-up time of prognosis, and patients' specificity. Our results showed that the PQC, CK-MB, and Cr were significantly different between the survival group and the death group. A large number of studies [28, 29] showed that the PQC was significantly associated with the prognosis of APP patients; our results also proved this point of view but, unfortunately, did not reach the same high correlation of prognosis compared with previous studies. PQ itself had direct nephrotoxicity; renal failure also impaired the excretion of PQ through the kidney; therefore, renal function injury may have a significant contribution to the mortality of APP [3]; the increase of Cr could suggest kidney injury [30]. CK-MB is the most specific and common indicator in the diagnosis of myocardial and skeletal muscle diseases, and a previous study that examined skeletal muscles obtained in both the biopsy and the autopsy of APP patients revealed extensive degeneration and fibrosis [3].

Compared with the single Rad-score, the nomogram that combined the clinical risk factors improved sensitivity, specificity, AUC, and diagnostic accuracy. The possible reason was that the CT image radiomics features mainly reflected the lung injury; by adding the clinical risk factors, the nomogram could reflect the damage of PQ to other tissues such as the liver, kidney, and muscle, so the performance of the model can be improved. However, the contribution of clinical risk factors was still lower than the radiomics signature, which indicated that the lung injury was the main prognostic factor in the early stage of poisoning.

In the previous studies about the mortality of APP patients, more attention was focused on lung nonspecific indicators. Many blood laboratory indicators were demonstrated to be useful in predicting the prognosis of patients with PQ poisoning [11, 14, 28]. These studies suggested that various laboratory indicators were related to prognosis in different degrees, but they all lacked independent validation. In a recent study [31], among 103 APP patients, aspartate aminotransferase, prothrombin time, prothrombin activity, total bilirubin, direct bilirubin, indirect bilirubin, alanine aminotransferase, urea nitrogen, and creatinine were found to be the most highly correlated indices in PQ poisoning and showed statistical significance (*P* < 0.05) in predicting PQ poisoning prognosis. Based on the above indicators, they established the grey wolf optimization-extreme learning machine (GWO-ELM) model. And the 10-fold cross-validation achieved a prediction accuracy of 81.45%, sensitivity of 81.24%, and specificity of 90.48%, respectively. Although the single-clinical factor model or multiclinical factor prediction model reached a certain accuracy, they were still lower than the prediction results of the Rad-score clinical model. This may be explained by two reasons; firstly, the baseline clinical data cannot specifically reveal the lung damage, which was the main cause of death; secondly, the data collection time was too early to fully reflect the damage of PQ toxicity to various organs. It was expected that lung CT images contained complementary and interchangeable information compared to other indexes, such as demographics, pathology, blood biomarkers, and genomics; combining the information would improve individualized treatment selection and monitoring [32].

This study has several limitations. Firstly, when choosing the ROI, mediastinal emphysema or pneumothorax and pleural effusion were not included; the main reason is that these signs may conceal the damage caused by PQ to the lung tissue, but the previous studies [6, 33] showed the appearance of mediastinal emphysema or pneumothorax, which suggested that the prognosis is bad and the mortality is high, so these signs' value of prognosis should be further studied. Secondly, in this study, the clinical risk factors of prognosis are not rich, such as urine PQ concentration, and arterial blood gas analysis was not included in the study, which was mainly restricted by hospital conditions. Whether there are significant differences in these clinical factors between the two groups and whether they can increase the performance of the prediction model need to be further discussed. Lastly, the relatively small sample number is another limitation of our study, which may have brought some deviation to the result, so it is necessary to make a further multicenter validation with a large number of samples in the future.

## 5. Conclusion

This study presents a radiomics nomogram that incorporates both the radiomics signature and the clinical risk factors and can be conveniently used to facilitate the individualized prediction of prognosis in patients with paraquat poisoning. Our study also proved that radiomics can also be applied to nontumor and diffuse diseases.

## Figures and Tables

**Table 1:** The demography and initial blood laboratory.

Factors		Survival group	Death group	*P* value	AUC
Age (years)		34.29 ± 10.98	37.61 ± 14.38	0.468	—
Gender	Male	25 (51%)	18(58.1%)	—	—
	Female	24 (49%)	13(41.9%)	—	—
PQC (mL)		3.84 (3.12)	8.60 (4.93)	0.000	0.804
WBC (×10^9^/L)		10.60 (4.15)	14.90 (8.30)	0.000	0.759
hsCRP (mg/L)		1.05 (3.78)	1.90 (6.70)	0.410	0.564
LDH (U/L)		205.50 (54.15)	228.55 (58.68)	0.031	0.698
CK-MB (U/L)		17.00 (5.83)	25.15 (15.23)	0.000	0.759
ALT (U/L)		16.55 (13.53)	19.15 (11.68)	0.253	0.562
AST (U/L)		19.95 (7.08)	22.35 (9.88)	0.277	0.550
ALB (g/L)		46.30 (4.53)	45.50 (8.95)	0.445	0.483
K (mmol/L)		3.71 (0.45)	3.56 (0.63)	0.091	0.387
Urea (mmol/L)		3.90 (2.40)	5.05 (2.10)	0.078	0.633
Cr (*μ*mol/L)		63.00 (19.20)	86.10 (41.00)	0.000	0.732
AMY (U/L)		109.50 (164.65)	92.00 (137.00)	0.698	0.468
GLU (mmol/L)		6.38 (1.69)	7.05 (2.24)	0.024	0.699

**Table 2:** The accuracy of the Rad-score.

Information	Rad-score		Nomogram	
	Train dataset	Validation dataset	Train dataset	Validation dataset
AUC (95%)	0.942 (0.886-0.997)	0.865 (0.658-1)	0.973 (0.936-1)	0.944 (0.844-1)
ACC	0.895	0.87	0.947	0.913
Specificity	0.914	0.929	0.955	0.929
Sensitivity	0.864	0.778	0.943	0.889
Threshold	0.358	0.358	0.607	0.607

## Data Availability

The data used to support the findings of this study are available from the corresponding author upon request.

## Supplementary Materials

Supplementary Materials Supplementary Materials provides the individualized comprehensive treatment plan, the inclusion and exclusion criteria, and the radiomics feature extraction methodology.
