# Supplementary material for: Development and Validation of a Radiomics Nomogram for Prognosis Prediction of Patients with Acute Paraquat Poisoning: A Retrospective Cohort Study
Source: Biomed Res Int. 2021 Feb 2;2021:6621894. doi: 10.1155/2021/6621894 (PMC7872759; doi:10.1155/2021/6621894)
Supplement: Supplementary Materials — provides the individualized comprehensive treatment plan, the inclusion and exclusion criteria, and the radiomics feature extraction methodology. [file 6621894.f1.docx]

**Development and Validation of a Radiomics Nomogram for Prognosis Prediction of Patients with Acute Paraquat Poisoning: a retrospective cohort study**

**Appendix A1:** **Individualized comprehensive treatment plan**

Individualized comprehensive treatment plan: Patients without gastric lavage in the local hospital were given gastric lavage as soon as possible, orally taken active carbon, magnesium sulfate and mannitol to clear poison, and given hemoperfusion combined with hemodialysis at the same time. The treatment plan also provided immunosuppression, cyclophosphamide and anti-oxidative damage drugs for patients, with appropriate rehydration and symptomatic supportive care. The treatment plan would be adjusted according to the changes of patients’ condition.

**Appendix A2:** **Inclusion and exclusion criteria**

Inclusion criteria for patients with paraquat poisoning:

1. Paraquat poisoning diagnosis was clear, the diagnosis consents the Chinese Medical Association emergency physicians branch "acute paraquat poisoning diagnosis and treatment expert consensus (2013)" paraquat poisoning diagnostic criteria;
2. came to the emergency department within 24 hours;、
3. Completed poison inspections, initial blood biochemical tests, and completed images and clinical data;
4. no significant other non-toxicity change in lung images.

Exclusion criteria included :

1. other drugs or pesticide poisoning ;
2. a history of severe lung disease such as persistent lung infection or recent infectious disease；
3. having past pulmonary tuberculosis, idiopathic pulmonary fibrosis or severe liver, kidney, blood system and other serious diseases;
4. lost follow-up within 30 days;
5. having been treated in our hospital for more than 24 h;
6. died prior to collecting clinical information or performing CT examinations.

**Appendix A3 Radiomics feature extraction methodology**

In our study, a total of 385 imaging features for each patient were extracted. All radiomics features were calculated automatically with the noncommercial A.K. (Analysis Kit) software (GE Healthcare, China). The 385 features were divided into three types: first-order: histogram parameters; second order texture features: gray-level co-occurrence matrix (GLCM), gray-level run length matrix (GLRL) features and gray-level size zone matrix (GLSZM) features and form factor features

1. **First-Order and Distribution Statistics:
   Minimum Intensity:** The value of the voxel(s) in the image ROI with the least value. **Maximum Intensity:** The value of the voxel(s) in the image ROI with the greatest value. **Mean Intensity:** The mean of the intensity or parameter values within the image ROI.


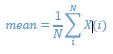


**Median Intensity:** The median of the intensity or parameter values within the image ROI. **Standard Deviation:** Measures the amount of variation or dispersion from the mean of the values in the image ROI.


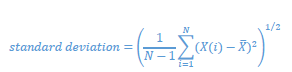


**Variance:** The mean of the squared distances of each value in the image ROI from the mean of the values. This is a measure of the spread of the distribution about the mean.


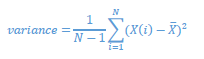


**Voxel Count:** The total number of voxels within the ROI of the grayscale image or parameter map. Describe the size of the ROI.

**Voxel Value Sum**: The sum of voxels within the ROI of the grayscale image or parameter map.

**Skewness:** Measures the asymmetry of the distribution of values in the image ROI about the mean of the values. Depending on where the tail is elongated and the mass of the distribution is concentrated, this value can be positive or negative.


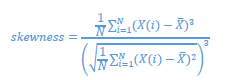

**Kurtosis:** A measure of the 'peakedness' of the distribution of values in the image ROI. A higher kurtosis implies that the mass of the distribution is concentrated towards the tail(s) rather than towards the mean. A lower kurtosis implies the reverse, that the mass of the distribution is concentrated towards a spike the mean.


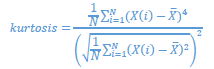


**Root Mean Square:** The square-root of the mean of the squares of the values in the image ROI. It is another measure of the magnitude of the image values.


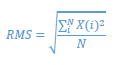


**Uniformity:** A measure of the sum of the squares of each discrete value in the image ROI. This is a measure of the heterogeneity of an image, where a greater uniformity implies a greater heterogeneity or a greater range of discrete image values.


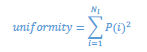


**Range:** The difference between the highest and lowest voxel values within the image ROI.

**Mean Deviation:** The mean of the distances of each image value from the mean of all the values in the image ROI.
**Energy:** A measure of the magnitude of values in an image. A greater amount larger values implies a greater sum of the squares of these values.


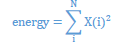

**Entropy:** Specifies the uncertainty in the image values. It measures the average amount of information required to encode the image values.


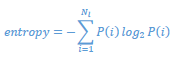


1. **Texture: Gray-Level Co-occurrence Matrix (GLCM)
   Energy (GLCM):** Also known as the Angular Second Moment and is a measure of the homogeneity of an image. A homogeneous image will contain less discrete gray levels, producing a GLCM with fewer but relatively greater values of P(i,j), and a greater sum of the squares.


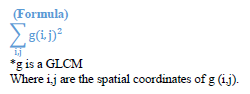

**Entropy(GLCM):** Indicates the uncertainty of the GLCM. It measures the average amount of information required to encode the image values.


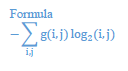

**Inertia:** It reflects the clarity of the image and texture groove depth. The contrast is proportional to the texture groove, high values of the groove produces more clarity, in contrast small values of the groove will result in small contrast and fuzzy image.


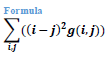
 **Correlation:** A value between 0 (uncorrelated) and 1 (perfectly correlated) showing the linear dependency of gray level values in the GLCM. For a symmetrical GLCM, ux= uy (means of px and py) and sigx = sigy (standard deviations of px and py).


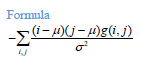

**Inverse Difference Moment Normalized (IDMN):** A measure of the local homogeneity of an image. IDMN weights are the inverse of the Contrast weights (decreasing exponentially from the diagonal i=j in the GLCM). Unlike Homogeneity 2, IDMN normalizes the square of the difference between values by dividing over the square of the total number of discrete values.


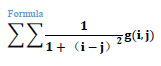

**Cluster Prominence:** A measure of the skewness and asymmetry of the GLCM. A higher values implies more asymmetry about the mean value while a lower value indicates a peak around the mean value and less variation about the mean.
**Cluster Shade:** A measure of the skewness and uniformity of the GLCM. A higher cluster shade implies greater asymmetry.
**Angular Second Moment:** ASM is a measure of homogeneity of an image. A homogeneous scene will contain only a few gray levels, giving a GLCM with only a few but relatively high values of P (i, j ). Thus, the sum of squares will be high.
**Contrast:** A measure of the local intensity variation, favoring P(i,j) values away from the diagonal (i != j), with a larger value correlating with larger image variation.
**Variance (GLCM):** The dispersion of the parameter values around the mean of the combinations of reference and neighborhood pixels, with values farther from the mean weighted higher. A high variance indicates greater distances of values from the
mean.
**Sum Average:
Sum Variance:** Weights elements that differ from the average value of the GLCM.
**Sum Entropy:
Difference Entropy:
Difference Variance:
(3)Texture: Gray-Level Run Length Matrix (GLRLM)**

The grey level run-length matrix (RLM) 𝐏𝐫(𝐢, 𝐣 | 𝛉 ) is defined as the numbers of runs with pixels of gray level *i* and run length *j* for a given direction θ. RLMs is generated for each sample image segment having directions (0°,45°,90° &135°), then the following ten statistical features were derived: short run emphasis, long run emphasis, grey level non-uniformity, run length non-uniformity, Low Grey Level Run Emphasis, High Grey Level Run Emphasis, Short Run Low Grey Level Emphasis, Short Run High Grey Level Emphasis, Long Run Low Grey Level Emphasis and Long Run High Grey Level Emphasis. **Short Run Emphasis (SRE):** A measure of the distribution of short run lengths, with a greater value indicative of shorter run lengths and more fine textural textures.


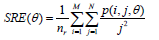

**Long Run Emphasis (LRE):** A measure of the distribution of long run lengths, with a greater value indicative of longer run lengths and more coarse structural textures.


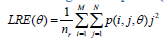

**Gray Level Non-Uniformity (GLN):** Measures the similarity of gray-level intensity values in the image, where a lower GLN value correlates with a greater similarity in intensity values.


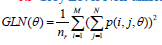

**Run Length Non-Uniformity (RLN):** Measures the similarity of run lengths throughout the image, with a lower value indicating more homogeneity among run lengths in the image.


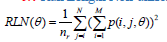

**Low Gray Level Run Emphasis (LGLRE):** Measures the distribution of low gray-level values, with a higher value indicating a greater concentration of low gray level values in the image.


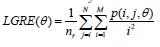

**High Gray Level Run Emphasis (HGLRE):** Measures the distribution of the higher gray-level values, with a higher value indicating a greater concentration of high gray-level values in the image.


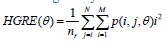

**Short Run Low Gray Level Emphasis (SRLGLE):** Measures the joint distribution of shorter run lengths with lower gray-level values.


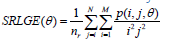

**Short Run High Gray Level Emphasis (SRHGLE):** Measures the joint distribution of shorter run lengths with higher gray-level values.


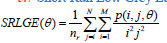

**Long Run Low Gray Level Emphasis (LRLGLE):** Measures the joint distribution of long run lengths with lower gray-level values.


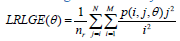

**Long Run High Gray Level Emphasis (LRHGLE):** Measures the joint distribution of long run lengths with higher gray-level values.


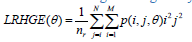


（4）**Texture: gray level Size Zone Matrix (SZM)**

The gray level Size Zone Matrix (SZM) is the starting point of Thibault matrices.[8] For a texture image f with N gray levels, it is denoted GSf(s, g) and provides a statistical representation by the estimation of a bivariate conditional probability density function of the image distribution values. It is calculated according to the pioneering Run Length Matrix principle: the value of the matrix GSf(s, g) is equal to the number of zones of size s and of gray level g. The resulting matrix has a fixed number of lines equal to N, the number of gray levels, and a dynamic number of columns, determined by the size of the largest zone as well as the size quantization.


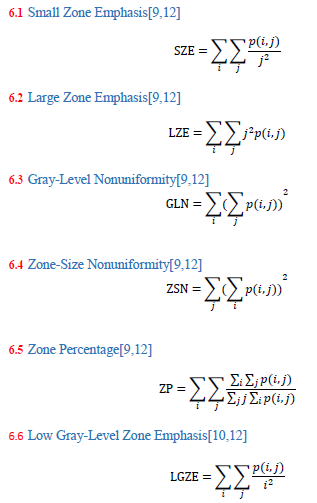


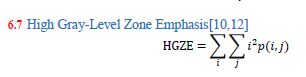


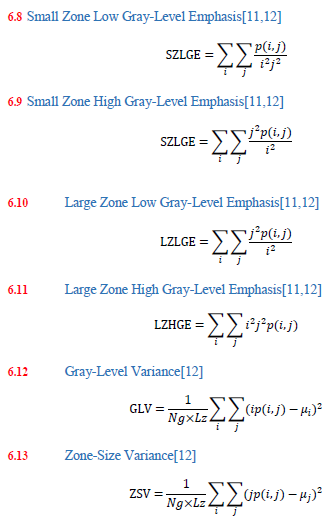


**（5）Form factor parameters**These group of features includes descriptors of the three-dimensional size and shape of the tumor region. Let in the following definitions 𝑉 denote the volume and 𝐴 the surface area of the volume of interest. We determined the following shape and size based features:


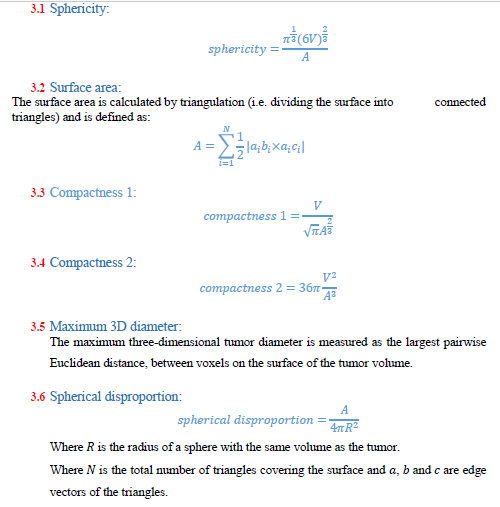


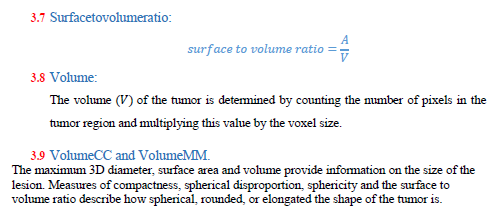


**Appendix Figure A1**





**Appendix Figure A1** Screen plot of PCA.

**Appendix Figure A2**







**Appendix Figure A2.** Rad-score for every patient in each cohort. Figure A2a.
Rad-score for every patient in the primary cohort; Figure A2b. Rad-score for every
patient in the validation cohort. The status of prognosis was marked with different
colors.
